# New World Hantaviruses Activate IFNλ Production in Type I IFN-Deficient Vero E6 Cells

**DOI:** 10.1371/journal.pone.0011159

**Published:** 2010-06-17

**Authors:** Joseph Prescott, Pamela Hall, Mariana Acuna-Retamar, Chunyan Ye, Marc G. Wathelet, Hideki Ebihara, Heinz Feldmann, Brian Hjelle

**Affiliations:** 1 Laboratory of Virology, Division of Intramural Research, National Institute of Allergy and Infectious Disease, National Institutes of Health, Rocky Mountain Laboratories, Hamilton, Montana, United States of America; 2 Research Service (151), New Mexico Veterans Affairs Health Care System, Albuquerque, New Mexico, United States of America; 3 Department of Pathology, Center for Infectious Diseases and Immunity, School of Medicine, University of New Mexico, Albuquerque, New Mexico, United States of America; 4 Department of Biology, Center for Infectious Diseases and Immunity, School of Medicine, University of New Mexico, Albuquerque, New Mexico, United States of America; 5 Department of Molecular Genetics and Microbiology, Center for Infectious Diseases and Immunity, School of Medicine, University of New Mexico, Albuquerque, New Mexico, United States of America; 6 Infectious Disease Program, Lovelace Respiratory Research Institute, Albuquerque, New Mexico, United States of America; Institut Pasteur, France

## Abstract

**Background:**

Hantaviruses indigenous to the New World are the etiologic agents of hantavirus cardiopulmonary syndrome (HCPS). These viruses induce a strong interferon-stimulated gene (ISG) response in human endothelial cells. African green monkey-derived Vero E6 cells are used to propagate hantaviruses as well as many other viruses. The utility of the Vero E6 cell line for virus production is thought to owe to their lack of genes encoding type I interferons (IFN), rendering them unable to mount an efficient innate immune response to virus infection. Interferon λ, a more recently characterized type III IFN, is transcriptionally controlled much like the type I IFNs, and activates the innate immune system in a similar manner.

**Methodology/Principal Findings:**

We show that Vero E6 cells respond to hantavirus infection by secreting abundant IFNλ. Three New World hantaviruses were similarly able to induce IFNλ expression in this cell line. The IFNλ contained within virus preparations generated with Vero E6 cells independently activates ISGs when used to infect several non-endothelial cell lines, whereas innate immune responses by endothelial cells are specifically due to viral infection. We show further that Sin Nombre virus replicates to high titer in human hepatoma cells (Huh7) without inducing ISGs.

**Conclusions/Significance:**

Herein we report that Vero E6 cells respond to viral infection with a highly active antiviral response, including secretion of abundant IFNλ. This cytokine is biologically active, and when contained within viral preparations and presented to human epithelioid cell lines, results in the robust activation of innate immune responses. We also show that both Huh7 and A549 cell lines do not respond to hantavirus infection, confirming that the cytoplasmic RNA helicase pathways possessed by these cells are not involved in hantavirus recognition. We demonstrate that Vero E6 actively respond to virus infection and inhibiting IFNλ production in these cells might increase their utility for virus propagation.

## Introduction

Vero E6 cells are epithelioid cells derived from the kidney of African green monkeys and, with their permissiveness for high viral productivity, are preferred for the isolation and/or propagation of the members of several virus families, including hantaviruses (a genus of the family *Bunyaviridae*) [1], [2], [3], [4], [5], [6], [7], [8]. The utility of these cells for virus propagation is at least three-fold. First, they are infectable by several diverse viruses, perhaps due to an abundant receptor repertoire, or non-specific uptake of virus particles. Second, they have sustained a genetic deletion that ablated the type I IFN locus [9]. Third, Vero E6-encoded interferon regulatory factor 3 (IRF3), a transcription factor necessary for generating responses to virus infection, is relatively inefficient, resulting in a muted initial response to virus infection [10]. For experimental purposes, hantavirus infectious stocks typically consist of the supernatants of infected Vero E6 cells and are used for *in vivo* and *in vitro* experiments.

At the cellular level, virus infection is detected by interactions between host-encoded pattern recognition receptors (PRRs) and pathogen-associated molecular patterns (PAMPs). Receptor binding activates several well-characterized signaling cascades and results in the activation of type I IFN. More recently, type III IFNs (IFNλ) have been described [11]. These IFNs are comprised of three genes that encode proteins for IFNλ1 (IL29), IFNλ2 (IL28A) and IFNλ3 (IL28B). IFNλ expression is controlled by pattern recognition receptor PRR activation, which activates and mobilizes IRF3 and IRF7, which in turn bind to the interferon-stimulated response elements (ISREs) of these genes, along with several other genes. IFNλs signal through a heterodimeric cell surface receptor comprised of IFNLR1 and IL10R2 [12], [13]. Expression of the IFNLR1 is cell-type specific, with high expression in epithelial cells and little or no expression in endothelial and fibroblast cells, rendering the latter unresponsive to the effects of IFNλ [14], [15]. Receptor engagement converges with that of the type I IFN receptors in that both activate the Jak/STAT signaling pathway leading to the formation of ISGF3 (STAT1/STAT2/IRF9), a transcription factor that regulates the expression of several ISGs, including MxA [16], [17]. Therefore, both the expression and function of IFNλs is much the same as for the type I IFNs.

Sin Nombre virus (SNV) and Andes virus (ANDV) are the most important agents of hantavirus cardiopulmonary syndrome (HCPS) in North America and South America, respectively [7], [18], [19]. HCPS is characterized by pulmonary edema due to capillary leak, followed by cardiogenic shock. Approximately 500 cases by SNV-related viruses have been reported in the U.S. since 1993 with a 32% case-fatality ratio (www.cdc.gov/ncidod/diseases/hanta/hps/index.htm). These viruses are carried by distinct rodent hosts and transmitted to humans by inhalation of virus-contaminated urine, feces and/or saliva. [20]. Vascular endothelial cells are thought to be the major site of viral replication in humans, and infected cells secrete high levels of chemokines and cytokines as a result [21], [22], [23], [24]. As in many viral systems, it appears that pathogenic hantaviruses possess mechanisms to antagonize the innate immune system and this antagonism has been hypothesized to play a role in the pathogenic process [25], [26], [27], [28]. For example, Prospect Hill virus (PHV), a New World hantavirus which has not been identified as a human pathogen, induces a strong IFN response, whereas the induction by ANDV is much weaker, likely correlating with their IFN antagonism efficiencies and possibly their pathogenic potential [29]. Recent work by our laboratory, as well as others, has focused on the mechanisms of PAMP-PRR engagement and the processes involved in antiviral activities and IFN antagonism [3], [29], [30], [31], [32], [33], [34], [35]. Many of these studies have also used human cell lines to characterize these interactions, including Huh7, a hepatoma cell line, as well as A549, a lung carcinoma cell line. Both of these cell lines are deficient in the TLR3 PRR axis but support hantavirus replication [3], [34], [36], [37].

We have previously demonstrated that SNV can induce a strong innate immune response in human umbilical vein endothelial cells (HUVEC) as well as Huh7 cells. We observed several differences in innate immune activation upon infection with hantaviruses. For example, we observed a strong innate response in Huh7 despite blocking virus entry, which was not the observation in HUVEC [33], [34]. Therefore, we hypothesized the recognition mechanisms differ between endothelial cells and these cell lines and that a viral surface protein or a soluble mediator of the innate immune response may be responsible for innate immune stimulation. In this study we found that Vero E6 cells mount an innate immune response and secrete IFNλ following infection by several hantaviruses, and were able to show that Vero E6-derived IFNλ is solely responsible for the induction of ISGs in both Huh7 and A549 cells, whereas ISG stimulation in primary human endothelial cells is virus-specific. This is the first report demonstrating that Vero E6 cells secrete IFNλ in response to viral infection and this response can in turn elicit downstream biological processes.

## Results

### SNV stock virus generated in Vero E6 cells activates a transient ISG response in Huh7 independent of virus titer, replication or intact particles

**Figure 1:**
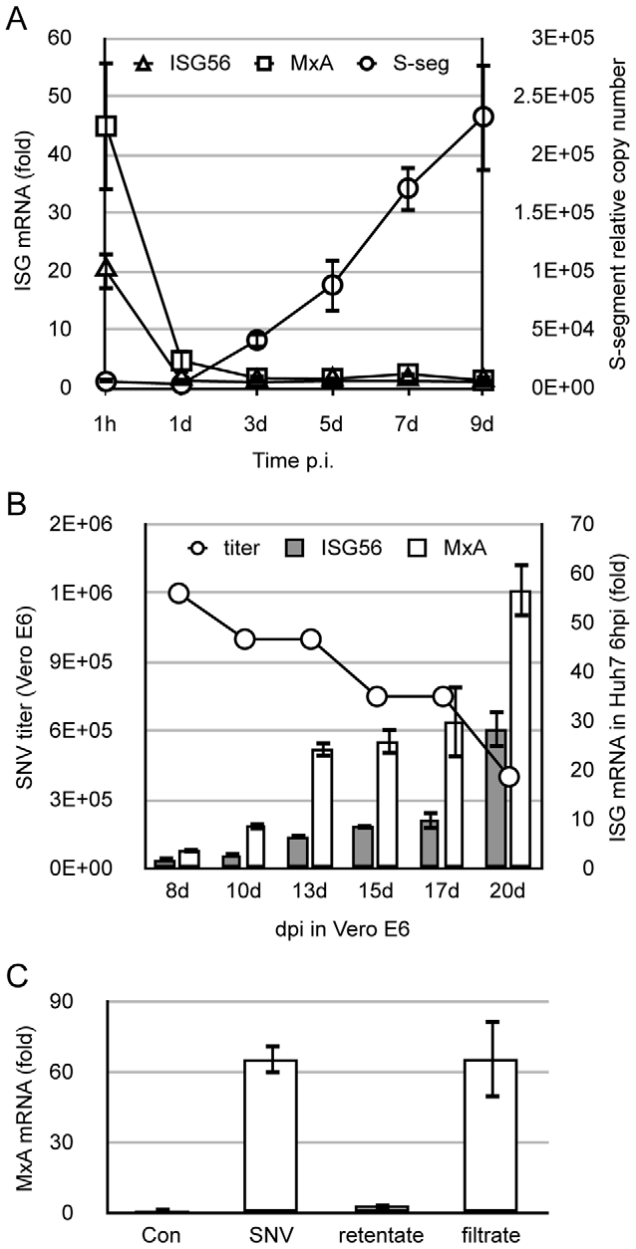
SNV stocks induce innate responses in Huh7 independent of titer, replication, and intact virus particles. (A) Huh7 cells were infected with SNV at an MOI = 1 for 1 h. At the time points indicated, cellular mRNA was isolated and used to quantitate viral S-segment RNA (right y-axis) and ISG expression (left y-axis) using qRT-PCR. (B) Vero E6 cells grown in 75 cm2 tissue culture flasks were inoculated with SNV (10 ffu) for 1 h and supernatants were collected at the time points indicated on the x-axis. Virus was then titrated on fresh Vero E6 cells (left y-axis) or used to infect Huh7 cells at equal volumes. ISG56 and MxA expression was measured by qRT-PCR 3 hpi (right y-axis). (C) SNV from 20 dpi Vero E6 stocks was applied to a 100-kDa filter and centrifuged for 10 min. The retentate and filtrate was resuspended to the loading volume and applied to Huh7 cells for 3 h. MxA expression was then measured using by qRT-PCR of total cellular RNA and compared to untreated control (Con) cells. All real-time qRT-PCR results are reported as the mean ± SEM from triplicate biological experiments.

We have previously demonstrated that, although both primary endothelial cells and the hepatoma cell line Huh7 respond to infection by hantaviruses by up-regulating ISGs, we observed several differences in cellular entry and transcription factor requirements that suggested a divergent PAMP and/or PRR axis had been activated in these distinct cell types [33], [34]. Because ISG inductions were observed in Huh7 in response to SNV stocks independent of entry and infection, we hypothesized that the ISG-activating component of the viral stocks may not be associated with infectious virus or the viral particle itself, but may be a soluble component derived from either the virus, or the Vero E6 cells used to propagate the virus. To test our hypothesis, we examined the kinetics of ISG56 and MxA activation by live SNV and compared this to viral replication kinetics in Huh7 cells. SNV induced a strong ISG response upon initial infection, however, this response was transient and almost completely abated by one day post infection (dpi), despite the continuous accumulation of viral S-segment vRNA in Huh7 cells (Figure 1A). We verified that Huh7 cells were productively infected, since we were also able to use their supernatants to infect fresh Vero E6 cells and focus assays performed on Vero E6 cells showed titers of up to 10^4^ ffu/mL (data not shown). These results indicate that either the products of viral replication are not required for ISG stimulation, or that the virus can efficiently antagonize innate immune responses following replication, thus inhibiting ISG activation. We then asked whether the ISG-activating potential in Huh7 is a function of viral titer upon initial infection. For this, we generated a time-course series of SNV stocks in Vero E6 cells and assayed both their viral titer on fresh Vero E6 cells and their ISG-inducing potential on Huh7 cells (Figure 1B). We observed that ISG induction is dissociable from the viral titer, with the highest ISG-inducing activity exhibited by the 20 dpi virus stocks, which had the lowest viral titers. To test whether a soluble mediator of innate immunity is responsible for the observed ISG transcription, we examined whether the ISG-activating fraction is physically dissociable from intact viral particles by filtration. We applied SNV stocks to a 100-kDa cut-off Amicon centrifugation filters and collected the filtrate and retentate, both of which we resuspended to the starting volume by the addition of cell culture medium. We were unable to detect replication-competent virus in the filtrate by titration (data not shown). When these components were applied to Huh7 cells, we observed that the ISG activating fraction was contained within the filtrate, indicating that a soluble component derived from the Vero E6 cell stocks, or viral components of less than 100-kDa relative mass are responsible for ISG inductions (Figure 1C).

### Vero E6 cells secrete IFNλ in response to SNV

**Figure 2:**
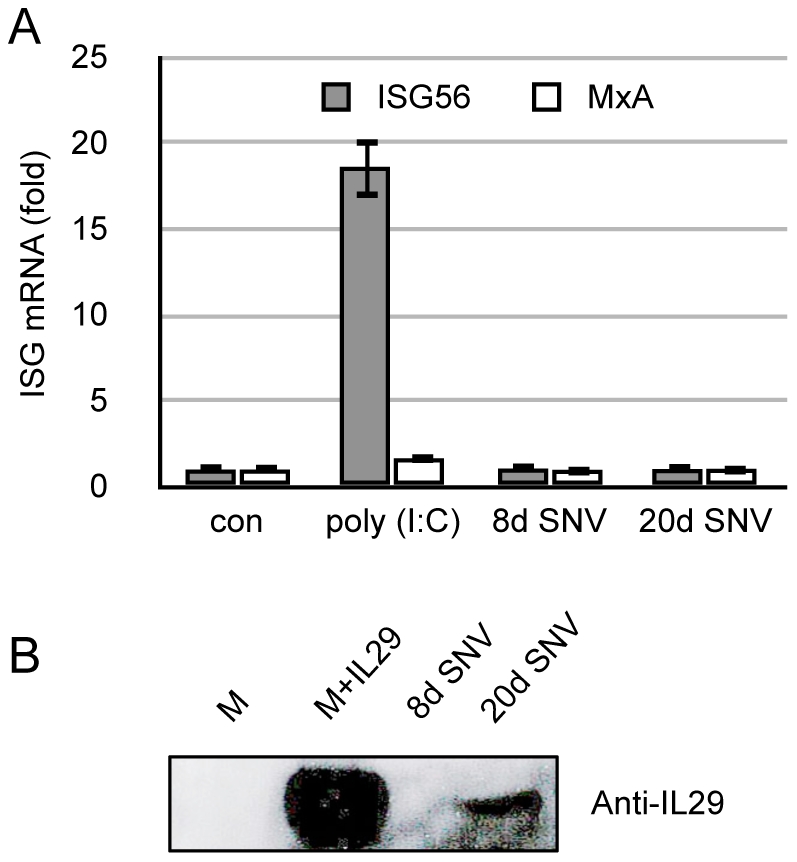
Vero E6-derived SNV stocks induce innate responses via a Jak/STAT pathway and contain IFNλ. (A) Hec 1b Jak/STAT knockout cells were treated with poly (I∶C) (50 µg/mL) or the indicated stock of virus from Figure 1 at an MOI = 1 for 3 h. Total cellular RNA was isolated and used for qRT-PCR for ISG56 and MxA. Real-time qRT-PCR results are reported as the mean ± SEM from triplicate biological experiments. (B) Medium supplemented with 50 µg/mL of rhIL29, and SNV derived from Vero E6 8 dpi and 20 dpi were applied to a 100-kDa filter and centrifuged for 10 min. The filtrate was then applied to a 3-kDa filter and centrifuged for 30 min. Equal volumes of the retentate were then used for SDS-PAGE. Western blotting was then performed using an anti-IL29 antibody.

We screened several human cell lines for their ability to activate ISG56 and MxA in response to SNV. In most cases, adherent human cell lines were able to respond, as assessed by their strong and early induction of expression of these genes. An exception to this was a HEC 1b cell line that is deficient in Jak/STAT signaling (kindly provided by Marc Wathelet). Poly (I∶C) (50 µg/mL) was able to induce a strong ISG56 response, but failed to induce MxA, which requires IFN signaling via Jak1/STAT (Figure 2A). SNV stocks failed to induce a response in these cells. Both type I and type III IFNs utilize the Jak1/STAT signaling pathway to exert their effects. Since Vero E6 cells are unable to express type I IFNs, we hypothesized that SNV-infected Vero E6 cells express type III IFNs. We performed western blots using the SNV stocks generated 8 dpi and 20 dpi in Vero E6 cells and concentrated on a 3-kDa filter, after first removing viral particles using a 100-kDa filter. As a positive control, we spiked Vero E6-conditioned medium with recombinant human IL29 (rhIL29) prior to concentration. We were able to detect a band of approximately 32 kDa, corresponding to the known size of IL29 (and the rhIL29 used) in the 20 dpi sample, as well as the positive control, but not in Vero E6-conditioned medium alone or in the 8 dpi sample (Figure 2B), which is consistent with their ISG-inducing potential (Figure 1B).

To test whether the measured titer of SNV from Vero cells is falsely lowered by the presence of IFNλ in viral stocks, we conducted endpoint titers of virus in a series of twofold dilutions to determine whether the titers predicted by the more highly-diluted virus stocks appeared higher than those less diluted, on the assumption that an inhibitor such as IFNλ would become diluted and less and less effective in blocking focus production at higher dilutions. We observed that there was no such augmentation in apparent titer at higher dilution, indicating that routinely-measured titers are likely not affected by the presence of IFNλ (data not shown).

### Divergent hantaviruses induce IFNλ secretion in Vero E6 cells

**Figure 3:**
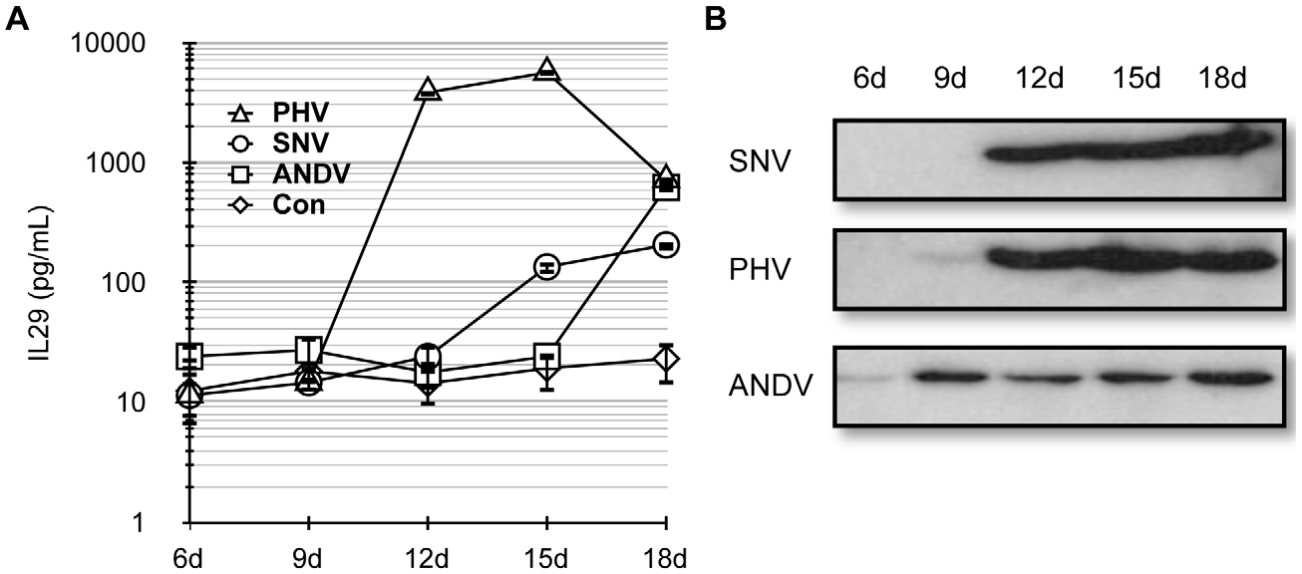
Divergent hantaviruses induce IFNλ in Vero E6 cells independent of virus replication. Vero E6 cells were grown in 75 cm^2^ culture flasks and infected with 10^3^ ffu of PHV, SNV or ANDV for 1 h, or left untreated (Con). Supernatants were collected at the indicated time points post infection and total medium was replaced. (A) Equal volumes of supernatants were used in an ELISA to detect IL29. Error bars represent the standard deviation between duplicate ELISA experiments. (B) Equal volumes of supernatants were used for SDS-PAGE followed by western blotting. Membranes were probed with an anti-hantavirus N antibody.

We asked whether IFNλ secretion by Vero E6 cells is specifically induced by SNV, or if IFNλ expression is a general feature of infection by hantaviruses. We compared the cellular IFNλ response to Prospect Hill virus (PHV), a New World hantavirus thought to be non-pathogenic, to SNV and Andes virus (ANDV), the primary etiologic agents of HCPS in the New World. Vero E6 monolayers were infected for equal amounts of hantavirus (1×10^3^ ffu) and supernatants were collected at the indicated time points and used for the detection of IFNλ by ELISA or for virus infection by western blotting of nucleocapsid (N). PHV induced a robust IFNλ response by 12 dpi and both ANDV and SNV induced modest levels of IFNλ at late time points post infection (Figure 3A). Mock-infected cultures had low levels of IFNλ for all time points, indicating that the increase in IFNλ secretion is not a result of prolonged cell culture or cell death at late time points, but is virus-specific. This method for generating hantavirus stocks using Vero E6 cells is typical and the IFNλ measured in these virus preparations represents the absolute amount of cytokine contained within a milliliter of a given virus stock. The low level of IL29 detection in the control samples is likely due to our substitution of a polyclonal detection antibody to achieve greater cross reactivity to the African green monkey-derived cytokine. Western blotting showed an accumulation of N protein in the supernatants, confirming that Vero E6 cells were productively infected by all three viruses (Figure 3B).

### Activation of ISGs by SNV stocks in Huh7 and A549 is due to IFNλ

**Figure 4:**
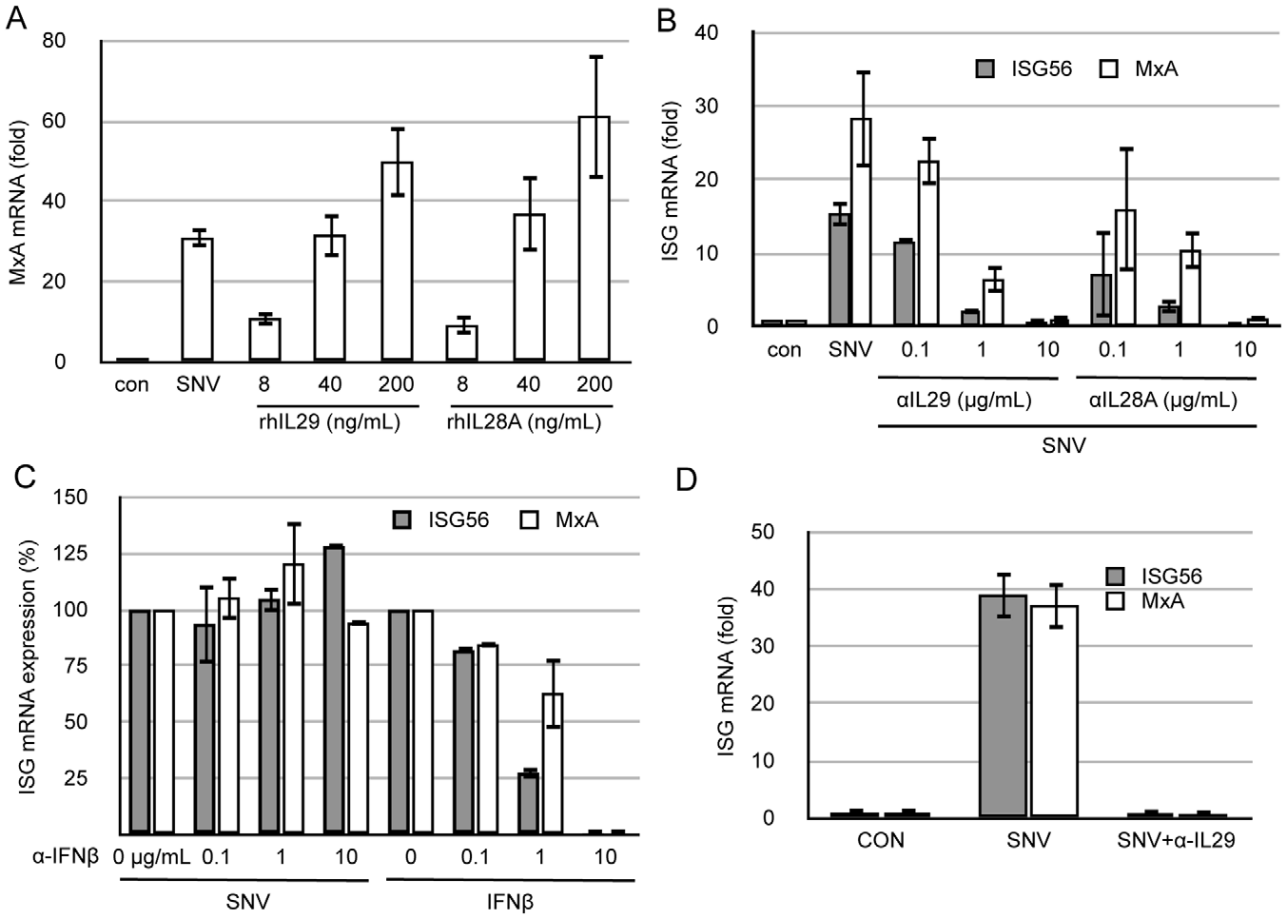
Innate responses to SNV in Huh7 and A549 are specifically due to Vero E6-derived IFNλ. Total cellular RNA was isolated from Huh7 or A549 cells and used for ISG quantitation by qRT-PCR. (A) Huh7 were treated with SNV (50 µL of virus) or the indicated amount of recombinant human IL29 or IL28A for 3 h prior to MxA mRNA quantitation by qRT-PCR. (B) Huh7 were infected with SNV at an MOI = 1 (50 µL) with or without prior incubation with anti-IL29 or anti-IL28A antibodies at the indicated final concentrations for 1 h. Three hours post infection, ISG56 and MxA mRNA expression was measured by qRT-PCR. (C) SNV or IFNβ was preincubated with the indicated amount of anti-IFNβ antibody for 1 h. Virus or IFNβ was then added to Huh7 cells for 3 h and ISG56 and MxA was measured 3 h post exposure by qRT-PCR. (D) A549 were infected with SNV at an MOI = 1, with or without prior incubation with anti-IL29 blocking antibodies (10 µg/mL). Three hours post infection ISG56 and MxA levels were measured by qRT-PCR. Real-time qRT-PCR results for all experiments (parts A–D) are reported as the mean ± SEM from triplicate biological experiments.

Type III IFNs are potent stimulators of the innate immune response. We suspected that the initial ISG response in Huh7 cells exposed to SNV stocks (Figure 1A) is due to Vero E6 cell-derived IFNλ. We assayed whether recombinant type III IFNs were able to induce an ISG response in Huh7 similar to that observed by our SNV stocks. Both IL29 (IFNλ1) and IL28A (IFNλ2) induced a strong, dose-dependent, MxA response in these cells (Figure 4A). We then analyzed the ability of anti-IL29 or anti-IL28A neutralizing antibodies to block ISG responses by pre-incubation with our viral stocks (MOI = 1, corresponding to 50uL of viral stock). Both neutralizing antibodies were able to efficiently inhibit ISG56 and MxA gene inductions in a dose-dependent manner (Figure 4B). Because both anti-IL29 and anti-IL28A antibodies blocked ISG inductions almost completely at the highest concentrations used (10 µg/mL), we assayed whether these antibodies cross react by blocking recombinant IL28A and IL29 respectively, and found that both antibodies cross reacted well with exogenous IFNλ proteins as measured by their ability to neutralize ISG activation in Huh7 cells (data not shown). As a control for antibody specificity and type III IFN sensitivity, we used recombinant IFNβ along with anti-IFNβ neutralizing antibodies and observed that although recombinant IFNβ induces a robust ISG response, the anti-IFNβ neutralizing antibody only inhibited recombinant IFNβ-mediated ISG responses, and not inductions by the SNV stock containing IFNλ, indicating that the anti-IFN antibodies are neutralizing only their homologous targets (Figure 4C). Along with Huh7, A549 cells are commonly used in hantavirus research and this human lung carcinoma cell line has been used to study innate immune responses in the context of infection by hantavirus [3], [35], [38], [39]. We asked whether this cell type is able to respond to SNV particles themselves, or whether ISG responses in A549 cells are attributed to the IFNλ contained within Vero E6-generated stocks. Similar to Huh7 cells, pre-incubation of SNV stocks with anti-IFNλ antibodies completely inhibited ISG responses observed in A549 when exposed to SNV particles at an early time point (Figure 4D).

### HUVEC respond specifically to SNV but not to IFNλ

**Figure 5:**
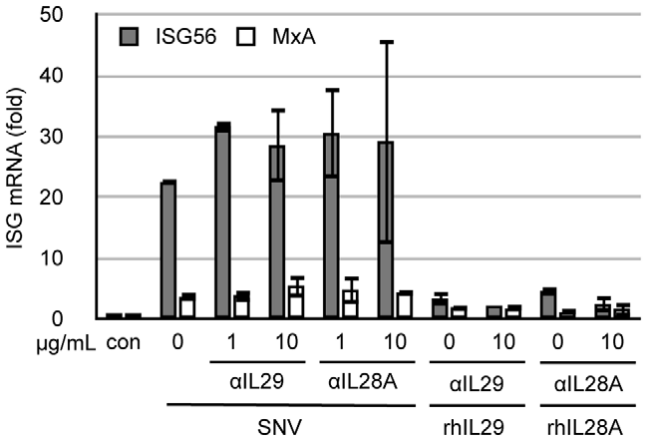
Endothelial cells specifically respond to SNV and are refractory to IFNλ. HUVEC were treated with SNV at an MOI = 1 (50 µL of stock virus), rhIL29 (50 ng/mL), rhIL28A (50 ng/mL), or left untreated (con) with or without pre-incubation with the indicated neutralizing antibody for 1 h. Cellular RNA was used to quantitate ISG expression 3 h post exposure. Real-time qRT-PCR results are reported as the mean ± SEM from triplicate biological experiments.

Endothelial cells are major target of hantaviruses *in vivo* and lack the IL28Rα receptor, which renders them incapable of responding to IFNλ [14]. We, as well as other groups, have reported that primary endothelial cells respond to hantavirus infection by up-regulating ISGs. We asked whether IFNλ contained within our viral stocks might play a role in ISG responses in HUVEC as they do in Huh7 and A549. We found that HUVEC transcribe only very low levels of ISG56 and MxA when exposed to either rhIL29 or rhIL28A. Furthermore, pre-incubation of SNV stocks with IL29 and IL28A neutralizing antibodies at doses shown to block IFNλ-mediated ISG induction in Huh7 and A549 cell lines, did not inhibit their ability to respond to SNV (Figure 5). This is consistent with our previous observations that HUVEC responses to SNV are specific, as SNV-specific neutralizing antibodies completely blocks ISG responses to virus [33].

## Discussion

Herein, we report that Vero E6 cells can recognize viruses and respond by secreting IFNλ. The Vero E6 cell line lacks the ability to transcribe type I IFNs as they possess a genetic deletion that ablates the entire type I IFN gene complex on both chromosomes [9]. In addition, it has recently been shown that these cells mount a weak initial IRF3-dependent response to various ligands, which partially accounts for their overall weak innate response to viruses and causes them to be extraordinarily competent for propagating several virus types to high titers [10]. Type III IFNs are transcribed independently of type I IFNs, and their activities are conferred through a different heterodimeric receptor, although both IFNs activate Jak/STAT signaling pathways and therefore their expression results in the transcription of a similar subset of ISGs. The quantity of IFNλ thus produced is sufficient to have a dramatic biological effect upon human cells. Different hantaviruses elicit different levels of expression of IFNλ, although all of the viruses that we tested induced the secretion of biologically active IFNλ in Vero E6 cells.

Hantaviruses primarily infect endothelial cells of the microvasculature and recent advances in the understanding of the innate immune response have prompted laboratories to study early events in viral recognition and host gene expression. Because primary cells are difficult to manipulate, several labs, including ours, have turned to established cell lines for use as a more tractable system for studying viral recognition and innate immune responses to infection. Many of these cell lines are of epithelial origin and express receptors for IFNλ. As a consequence, IFNλ secreted by Vero E6 cells used to generate viral stocks can cross species and dramatically alter innate gene expression. Herein, we demonstrate that this is true for both the Huh7 and A549 cell lines, which are the two cell lines most commonly used to study the interactions between hantaviruses and human host-cells in *in vitro* systems. We also confirm herein that innate immune studies performed using cells of endothelial origin are largely unaffected by IFNλ carryover from Vero E6 cells. These observations are in accordance with our previous studies showing that endothelial cell responses to hantaviruses vary according to viral serotype and rely on viral entry, in contrast to our observations regarding Huh7 cells [33], [34].

An unexpected observation was that Huh7 as well as A549 cells were completely unresponsive to SNV (as well as PHV and ANDV at early time points, data not shown) in that the viruses failed to elicit ISG activation. Blockage of signaling by Vero-derived IFNλ using antibodies that neutralize type III IFN completely abrogated the ISG response to virus stocks. Furthermore, despite continuous and high level of SNV viral replication, Huh7 cells never respond by upregulation of ISG56 or MxA, which are markers of activation of the IRF3 and Jak/STAT pathways respectively. These observations are in contrast to a recent report that Huh7 and A549 cells respond to hantaviruses by up regulating ISGs [3]. In the aforementioned study, both Huh7 and A549 cells mounted a strong and early MxA response when exposed PHV as compared to Hantaan virus (HTNV). We observe that stocks of PHV (12dpi) contain high levels of IFNλ, whereas the SNV and ANDV stocks contain less IFNλ for a given time point. It is possible that in their system, undetected Vero E6-derrived IFNλ induced by PHV and contained within virus preparations might account for their finding that PHV induces a robust and early innate response when compared to HTNV.

We observe levels of IFN lambda in hantavirus preparations that are sufficient to activate an ISG response when presented to epithelioid cells at a volume corresponding to less than an MOI = 1. This MOI represents approximately 50uL of SNV stock when used to infect near-confluent cells grown in 24-well plate format, and a greater volume of virus stock for both ANDV and PHV, as these viruses typically grow to a lower titer. Interestingly, we observe that this IFNλ is highly stable, especially compared to the type I IFNs. We were able to detect high levels of IFNλ by ELISA following 5 megarads of gamma irradiation (Fig. 3). We also observe little or no decline in the biological activity of IFNλ following UV-inactivation of virus preparations, one freeze-thaw cycle, or storage at 4C for up to one month (data not shown).

The lack of innate immune responsiveness to hantaviruses, independent of IFNλ, by Huh7 cells suggests that the PRR responsible for recognition of these hantaviruses, at least for SNV, is not RIG-I or Mda5, confirming our previous observations [34]. Huh7 cells, along with A549 cells possess intact RNA helicase pathways, but are unable to mount a TLR3-mediated response to several high titer replicating viruses [36], [37]. It is unlikely that this unresponsiveness presented herein is attributed to a virus-encoded interferon antagonist that is efficient enough to completely suppress ISG transcription, as this is not the observation when using endothelial cells. In support of our evidence that RIG-I is not the PRR used to detect hantaviruses, recent work has shown that RIG-I recognizes RNAs with 5′ triphosphate groups [40]. In this study, HTNV was shown to be sensitive to a 5′ monophosphate-specific exonuclease, suggesting a post-transcriptional modification of the RNA might be a strategy used by hantaviruses to avoid recognition by RIG-I [40]. Our data also suggests that Vero E6 cells possess an intact hantavirus-sensing PRR and IRF3 pathway. The delayed and prolonged IFNλ activation that we observe is likely due to the recently characterized weak endogenous Vero E6 IRF3 transcription factor as well as an impaired positive feedback amplification of the innate response due to the lack of type I IFNs [10]. These inherent characteristics, taken together with their ability to mount a type III IFN response, might lend Vero E6 cells particularly useful for examining the signaling events of early innate responses, and IFN antagonism, by hantaviruses, as well as other virus families.

Our finding that PHV can induce a stronger and earlier IFNλ response in Vero E6 cells is not surprising. HCPS-causing hantaviruses have been shown to induce a weak IFN response, whereas PHV induces IFNs much more readily [29]. This is likely due to an antagonistic capability of SNV and ANDV upstream of IFN activation. The isolate of ANDV we are using consistently replicates to lower titers than does the SN77734 isolate of SNV, although here we show that both viruses activate a similar IFNλ profile. This is possibly due to individual differences in their antagonistic potential in conjunction with their replicative fitness. Conversely, the profile of IFNλ induced by PHV in Vero E6 cells reinforces the observations that IFN antagonism by this virus is less efficient.

The modulation of IFNλ by hantaviruses may have clinically relevant implications. Interestingly, IFNλ expression has been observed to be important for human hantavirus infection *in vivo*. Patients infected with Puumala virus and presenting with nephropathia epidemica (NE) had significantly lower serum levels of IFNλ during acute infection compared with healthy control patients. *In vitro*, HTNV virus replication is inhibited in cells pretreated with IFNλ, although post infection treatment does not inhibit replication [38]. Furthermore, it has recently been shown that dendritic cells treated with IFNλ, but not type I IFNs, are able to polarize T cells to a Foxp3^+^ expressing tolerogenic phenotype, indicating that IFNλ might skew the immune system toward an anti-inflammatory response [41]. The natural host and reservoir for SNV is the deer mouse, which becomes persistently infected and displays little or no pathology [42]. CD4^+^ T cells from persistently infected deer mice display a Foxp3^+^ phenotype, indicative of T regulatory cells, and this may be a mechanism in which the deer mouse avoids an inflammatory-mediated pathogenic state [43]. The mechanism of the T cell regulation is currently unknown, but IFNλ might play a role in generating the observed suppressive response. The link between cell culture production of IFNλ and *in vivo* IFNλ expression, and its importance in the pathogenic process of hantavirus infection will need to be investigated further.

The activation of IFNλ by Vero E6 cells demonstrates that these cells mount an innate immune response to viral infection. The IFNλ response in Vero E6 is likely delayed because of their inherently weak IRF3 response and lack of type I IFN genes. This ISG response almost certainly affects the quality and quantity of viral stocks generated in this system. The onset of IFNλ production in response to hantaviruses correlates with a decrease in viral titers for all viruses tested, suggesting that these cells enter an anti-viral state, albeit inefficiently. Disruption of this innate response may render these cell types more efficient vessels for propagating virus stocks. This work has implications for all systems utilizing Vero E6-derived virus preparations and furthers the field of innate immunity to hantaviruses.

## Materials and Methods

### Cells, viruses and reagents

We purchased human hepatoma cells (Huh7) from Apath (New York). A549 and Vero E6 cells were purchased from ATCC. We grew cells in DMEM (Invitrogen) containing 10% FCS, 1X essential amino acids (Gibco), pen/strep, and gentamicin. We purchased pooled primary human umbilical vein endothelial cells (HUVEC), from Clonetics (San Diego, CA) and propagated them in EBM-2 medium supplemented with the Clonetics bullet kit, which supplies growth factors including vascular endothelial growth factor (VEGF). We previously described the isolation of Sin Nombre virus (SNV) strain SN77734 from a New Mexico deer mouse [42]. We obtained Andes virus (ANDV) strain CHI-7931 from H. Galeno, Instituto Salud Publico, Santiago, Chile and Prospect Hill virus (PHV) strain PH-1 from R. Yanagihara (Univ. of Hawaii). Hantaviruses were propagated and titrated on Vero E6 cells (ATCC) under strict standard operating procedures using biosafety level 3 (BSL3) facilities and practices (CDC registration number C20041018-0267).

For virus infections, we seeded cells in 48- or 24-well plates at a density to achieve 90–95% confluence after overnight incubation at 37°C in 5% CO_2_. Treatment was done essentially as described previously [33].

For antibody blocking studies, we incubated viruses or recombinant proteins with the indicated concentrations of anti-IL29, anti-IL28A, or anti-IFNβ (R&D Systems, AF1598, AF1587 or AF814, respectively) for 0.5 h at room temp. The resulting mixture was then added to cells and incubated for 3 h prior to performing the indicated assays. Recombinant proteins used included rhIL29, rhIL28A and rhIFNβ (R&D Systems, 1598-IL-025, 1587-IL-025 and 11415-1, respectively).

For poly (I∶C) treatment, we seeded cells as above and at the time of infection, and exposed cells to 10% DMEM containing 50 µg/mL poly (I∶C) (Sigma).

### Virus titrations

We infected monolayers of Vero E6 cells grown in 24-well plates with 200 µL of serial 10-fold diluted virus for 2 h. We then removed the inoculum and overlaid the cells with a 1.2% solution of methylcellulose in a final concentration of 1X DMEM, 2.5% FBS. The cells were incubated for seven days prior to staining for foci using a rabbit polyclonal anti-N antibody followed by the addition of a 1∶1000 dilution of an HRP-conjugated anti-rabbit- antibody (Jackson ImmunoResearch). Foci were developed using a metal-enhanced DAB substrate kit (Thermo Scientific).

### Real-Time SYBR Green and TaqMan RT-PCR

For SYBR Green RT-PCR, we performed reverse transcription using 2 µg of total RNA extracted from cell cultures using the RNeasy kit (Qiagen) in 40 µL reactions using the Applied Biosystems TaqMan Reverse Transcription reagents kit (ABI, Foster City, CA). For PCR, we used the Applied Biosystems Power SYBR Green reagents kit to perform reactions in triplicate using 3 µL of cDNA and a 0.4 µM final concentration of each primer in a 25 µL total reaction volume. The primers we used were specific for: β-actin, sense (5′-ccatcatgaagtgtgacgtgg-3′) and antisense (5′-gtccgcctagaagcatttgcg-3′); ISG56, sense (5′-tctcagaggagcctggctaag-3′) and antisense (5′-ccacactgtatttggtgtctagg-3′); MxA, sense (5′-tgatccagctgctgcatccc-3′) and antisense (5′-ggcgcaccttctcctcatac-3′). We calculated the fold-change for each gene using the mean of the change in CT values (ΔCT) normalized to the CT values of GAPDH for each sample (2^−ΔΔCt^) [33].

We performed TaqMan RT-PCR for SNV viral S-segment RNA as previously described [33]. We quantified the viral RNA using a standard curve generated using S-segment templates of known copy number.

### Supernatant filtration

We added 1 mL of the Vero E6-derived virus stocks to 100-kDa filtration units (Amicon) and centrifuged them at 4000×g for 15 min. The retentates and filtrates were resuspended to a final volume of 1 mL and equal volumes were added to Huh7 cells. For the samples used to detect IL29 by western blotting, we applied the filtrate of 100-kDa filter units to 3-kDa filter units (Amicon) and centrifuged them at 4000×g for 45 min. The retentate was then used directly in assays for ISG induction.

### Western blotting

For IL29 detection, we boiled the retentates of the 3-kDa filtration units along with a 4% SDS loading buffer for 5 min. We then separated retentates on a 12.5% SDS-PAGE gel and transferred the proteins to PVDF membranes. Membranes were then probed with a primary polyclonal rabbit anti-IL29 antibody (R&D Systems) overnight at 4C at a concentration of 0.2 µg/mL followed by an incubation with a 1∶10,000 dilution of a HRP-labeled anti-rabbit IgG secondary antibody for 1 h (Jackson ImmunoResearch). For hantavirus N detection, we used the methods described previously [34].

### ELISA of IL29

We performed ELISA using the human IL29 DuoSet ELISA Development System (R&D Systems, DY1598) as per their recommended protocol, with the exception of coating the plates with a polyclonal anti-human IL29 capture antibody (R&D Systems, AF1598). This antibody was used for its greater crossreactivity to African green monkey-derived IFNλ. We added 100 µL of Vero E6-derived viral stocks that had been subjected to 5 megarads of gamma irradiation, or Vero E6-conditioned medium as a control. The concentrations of IL29 were interpreted from a curve generated using 2-fold dilutions of a recombinant IL29 standard starting at 8000 pg/mL. All data were reported as the average of samples and standards run in duplicate wells.

**Statistical analysis** Statistical analyses were performed on replicate samples using an unpaired Student's *t* test. The mean ± standard error of the mean is represented and significance (*P*<0.05) is reported where appropriate.
